# Outcomes of lenvatinib therapy in poorly differentiated thyroid carcinoma

**DOI:** 10.1530/ETJ-23-0003

**Published:** 2023-03-28

**Authors:** João Roque, Tiago Nunes Silva, Catarina Regala, Ricardo Rodrigues, Valeriano Leite

**Affiliations:** 1 Endocrinology, Diabetes and Metabolism Department, Centro Hospitalar Universitário Lisboa Norte, Hospital de Santa Maria, Lisbon, Portugal; 2 Endocrinology, Diabetes and Metabolism Department, Instituto Português de Oncologia de Lisboa Francisco Gentil, Lisbon, Portugal; 3 NOVA Medical School | Faculdade de Ciências Médicas of Universidade NOVA de Lisboa, Lisbon, Portugal; 4 Unidade Investigação Patobiologia Molecular, Instituto Português de Oncologia de Lisboa Francisco Gentil, Lisbon, Portugal

**Keywords:** poorly differentiated thyroid carcinoma, lenvatinib, effectiveness, outcomes

## Abstract

**Background and objective:**

Lenvatinib showed promising results in a subgroup of patients with poorly differentiated thyroid carcinoma (PDTC) in the SELECT trial. Our aim was to report the effectiveness and tolerability of lenvatinib in our series of PDTC patients.

**Methods:**

Medical records of eight consecutive patients with PDTC treated with lenvatinib in a single center between January 2019 and October 2022 were retrospectively reviewed. Inclusion criteria were PDTC diagnosis based on Turin criteria and evidence of disease progression in the previous 6 months.

**Results:**

Eight PDTC patients received an average dose of lenvatinib of 18.1 mg for a median duration of treatment of 10.3 months. The baseline Eastern Cooperative Oncology Group performance status was ≥2 in 50% of patients. Two patients had unresectable primary tumor. Seven patients showed extrathyroidal disease, particularly mediastinal lymph nodes (85.7%), lung (71.4%), and bone (71.4%). The disease control rate was 100%, with partial response and stable disease in 12.5 and 87.5%, respectively. The median time to best overall response was 3 months, and the median duration of response was 7.5 months. Median progression-free survival was 12 months and median overall survival was not reached. At 6, 12, and 18 months, overall survival was 87.5, 71.4, and 57.1%, respectively. All patients experienced drug-related adverse effects (AEs). Four (50%) had dose reductions and two (25%) had temporary treatment interruptions. Lenvatinib was stopped in two patients due to grade ≥3 AEs.

**Conclusion:**

Lenvatinib is an effective treatment for real-world PDTC patients. Adequate management of comorbidities and AEs increases treatment tolerability and minimizes dose reductions.

## Introduction

The term poorly differentiated thyroid carcinoma (PDTC) was first introduced by Granner and colleagues in 1963, although it was only recognized as a distinct entity by the World Health Organization in 2004 (1, 2). In 2006, the Turin Criteria were published, establishing the current histologic criteria for PDTC diagnosis (1, 3). PDTC accounts for 3–5% of all thyroid carcinomas and corresponds to the most aggressive subset of differentiated thyroid carcinoma (DTC) (1, 4). At presentation, the majority of patients with PDTC have compressive symptoms with radiological evidence of extrathyroidal neck disease (1, 4). Distant metastasis eventually emerges during follow-up in up to 85% of cases, being the cause of most disease-related deaths (1). While DTC diagnosis can be usually established by fine-needle aspiration cytology, PDTC usually requires histological examination (4). Survival rates range between 50–85, 34–50, and 0% at 5, 10, and 15 years after diagnosis, respectively (1, 4, 5). Higher mortality rates have been associated with older age, primary tumor >4 cm, pT3/pT4 staging, and presence of distant metastasis (5). Therefore, more effective treatment alternatives will be of utmost importance for patients with PDTC. The available treatment modalities are the same as radioiodine (RAI)-refractory DTC, despite the absence of specific trials focusing on PDTC patients. Total thyroidectomy with lymph node dissection is the preferred treatment modality, with a significant impact on disease progression if all gross disease is removed (1, 4). Adjuvant treatment includes RAI, external beam radiotherapy (EBRT), and systemic chemotherapy. High RAI activities are recommended for patients with avid lesions. However, RAI avidity is quite variable in patients with PDTC, mainly due to the presence of less differentiated areas which decrease iodine-131 uptake and retention (1, 4). EBRT can be considered after R2 surgery, loco-regionally recurrent disease, or bone metastasis. Nevertheless, survival benefit has not been established (1, 6). Systemic cytotoxic chemotherapy has been used in a small number of cases without significant impact on the outcomes and with important side effects (1, 7). Recently, tyrosine kinase inhibitors (TKIs) have been used in PDTC patients enrolled in clinical trials targeting RAI-refractory DTC. To date, sorafenib and lenvatinib have been approved by the U.S. Food and Drug Administration and European Medical Agency for first-line treatment of RAI-refractory DTC (1). Cabozantinib has been used for second-line treatment (8). This was based on the significant improvement in progression-free survival (PFS), compared to placebo, obtained in the respective phase III trials, DECISION and SELECT (10.8 vs 5.8 months with sorafenib and 18.3 vs 3.6 months with lenvatinib) (1, 9, 10). Both studies included PDTC patients (11.6% for sorafenib and 10.7% for lenvatinib) (9, 10). Remarkably, lenvatinib elicited a PFS of 14.8 months (vs 2.1 with placebo) in this subgroup in the SELECT trial (10). Concerning cabozantinib, PDTC patients were considered in the inclusion criteria of COSMIC-311, but subgroup results are not available (8). Regarding tolerability, all grades and grade 3 or higher adverse effects (AEs) were reported in 97.3 and 75.9% of patients, respectively (10). Since the publication of SELECT results in 2015, real-world studies with RAI-refractory thyroid cancer patients treated with lenvatinib have been published and some have included PDTC patients, with encouraging results (Table 1) (11, 12, 13, 14, 15, 16, 17, 18). Based on these premises, we have retrospectively reviewed the effectiveness and tolerability of lenvatinib in patients with PDTC followed at our center.

**Table 1:** Real-world studies of lenvatinib therapy in RAI-refractory thyroid cancer which included a proportion of patients with PDTC.

Ref	Number of patients	PDTC (%)	ECOG 0/1/2+ (%)	Bone/lung metastasis (%)	Inoperable disease (%)	Previous TKI (%)	Initial/mean dose (mg)	PFS (months)
Porcelli *et al.* (2021) (13)	23	30.4	91 (1–2)/9	43.5/91.3	8.7	8.7	21/NA	25.0
Berdelou *et al.* (2018) (12)	75	25.3	NA/NA/16	58.7/88	4.0	68.0	21/20	10.0
Hamidi *et al.* (2022) (14)	27	22.2	22/74/4	51.9/92.6	18.5	3.7	20/NA	12.0
Kim *et al.* (2022) (17)	56	16.1	NA	7.1/53.6	0	0	20/NA	35.3
De Leo *et al.* (2021) (11)	13	15.4	62/38/0	15.4/38.5	0	0	20/11	22.0
Song *et al.* (2020) (18)	43	13.9	NA	58.1/95.3	0	74.4	20/10	21.8
Takahashi *et al.* (2020) (15)	442	12.7	51/36/13	37.3/87.9	5.2	NA	21/12	NA; time-to-treatment failure: 12.5
Jerkovich *et al.* (2020) (16)	22	4.5	37/45/18	45.4/72.7	NA	59.0	22/16	13.7

ECOG, Eastern Cooperative Oncology Group performance status; NA, not available/without access; PDTC, poorly differentiated thyroid carcinoma; PFS, progression-free survival; TKI, tyrosine kinase inhibitor.

## Methods

### Patients

We retrospectively analyzed the medical records of all adult patients with the diagnosis of PDTC treated with lenvatinib in the Endocrinology department of Instituto Português de Oncologia de Lisboa Francisco Gentil (IPOLFG) between January 2019 and October 2022. Our department is the referral center for most of the advanced thyroid cancer patients in Portugal. PDTC diagnosis was established according to the 2006 Turin Criteria. Informed consent for the treatment was obtained from all patients. Institutional treatment protocols were strictly followed, and the study was conducted in compliance with the Helsinki Declaration and was approved by IPOLFG Ethics Committee.

### Treatment and safety

Decision to start lenvatinib was made by a multidisciplinary team and followed evidence of progression in the last 6 months, either after surgery and adjuvant RAI or in progressive unresectable disease. Starting doses were defined according to patients’ characteristics, particularly age, Eastern Cooperative Oncology Group (ECOG) performance status scale (https://ecog-acrin.org/resources/ecog-performance-status/), and comorbidities. Whenever possible, lenvatinib was increased to the maximal tolerated dose. Also, dose reductions were performed based on tolerability and according to the manufacturer’s recommendations. Interruption or definitive suspension of treatment was considered when grade 3+ AEs occurred (according to Common Terminology Criteria for Adverse Events 5.0 (https://ctep.cancer.gov/protocoldevelopment/electronic_applications/ctc.htm) or in cases of persistent progression. Treatment of comorbidities was optimized before the initiation of lenvatinib. Clinical and biochemical follow-up was performed every month. All patients had a phone connection to the clinic for a prompt report of AEs and treatment.

### Effectiveness measures

Treatment effectiveness was assessed by computed tomography (CT), according to RECIST criteria 1.1 (19). CT scans were performed every 3 months. In selected cases, ^18^fluorodeoxyglucose positron emission/CT (^18^FDG-PET/CT) and magnetic resonance imaging were also used to evaluate progression. PFS, disease-specific survival (DSS), overall survival (OS), best overall response (BOR), disease control rate (DCR), and duration of response (DoR) were established as effectiveness outcomes. PFS was defined as the time between lenvatinib start and disease progression, death or last follow-up. OS and DSS were defined as the time between lenvatinib start and death by any cause or related to the disease, respectively. DoR was defined as the time between first evidence of response (stable disease, partial response, or complete response) and evidence of progression, death, or last follow-up.

### Statistical analysis

Continuous variables were analyzed through descriptive statistics and respective results were presented as mean with s.d. and median with interquartile range. Categorical variables were expressed as absolute numbers or percentages. Kaplan–Meier curves were used to calculate PFS, DSS, and OS. Statistical analyses were performed using SPSS^®^ (v.23) software.

## Results

### Patients’ characteristics at baseline

Baseline characteristics of the eight patients are summarized in Table 2. The median age at diagnosis was 59 years old and only one patient (12.5%) had more than 65 years old, with a female predominance. ECOG performance status was zero in one (12.5%), one in three (37.5%), and two in four (50%) patients. RAI treatment was performed in all six patients who were previously submitted to surgery, and the median total activity was 150 mCi. Four patients were also submitted to local EBRT and one to chemotherapy with paclitaxel and carboplatin. EBRT was also performed in bone metastatic lesions in three patients and surgical excision of metastasis was undertaken in two patients due to compressive symptoms. Treatment with lenvatinib was initiated either due to evidence of progression after previous treatments (six patients, 75%) or in the neo-adjuvant setting in patients with progressive inoperable disease (two patients, 25%). Loco-regional metastasis was present in four (50%) and distant metastasis was present in seven (87.5%) patients at lenvatinib start, particularly in mediastinal lymph nodes (six patients), lung (five patients), and bone (five patients).

**Table 2:** Baseline characteristics of the patients.

Characteristic	All patients (*n* = 8)
Median age at PDTC diagnosis, years (range)	59.0 IQR 10.3 (34–67)
Female, *n* (%)	5 (62.5)
ECOG Performance Status Scale at lenvatinib initiation, *n* (%)	
0	1 (12.5)
1	3 (37.5)
2	4 (50.0)
Median primary tumor longest diameter, mm (range)	56 IQR 20 (30–80)
Previous treatments, *n* (%)	
Surgery	6 (75.0)
RAI	6 (75.0)
Median total RAI activity, mCi (range)	150 IQR 227.5 (100–400)
Cervical ERBT	4 (50.0)
Chemotherapy	1 (12.5)
Tyrosine kinase inhibitor	3 (37.5)
Unresectable primary tumor, *n* (%)	2 (25.0)
Median age at lenvatinib start, years (range)	61.5 IQR 11.3 (36–72)
Median time from PDTC diagnosis to lenvatinib start, months (range)	19.0 IQR 66.0 (1–168)
Median time from radioiodine treatment to lenvatinib start, months (range)	36 IQR 84 (10–108)
Positive serum Tg at lenvatinib start, *n* (%)	5 (62.5)
Median serum Tg, ng/mL (range)	2865 IQR (879–17,842)
Loco-regional disease at lenvatinib start, *n* (%)	4 (50.0)
Distant metastasis at lenvatinib start, *n* (%)	7/8 (87.5)
Mediastinal lymph nodes	6/7 (85.7)
Lung	5/7 (71.4)
Bones	5/7 (71.4)
Liver	2/7 (28.6)
Skin	1/7 (14.3)

ECOG, Eastern Cooperative Oncology Group; ERBT, external beam radiotherapy; IQR, interquartile range; PDTC, poorly differentiated thyroid carcinoma; RAI, radioactive iodine; Tg, thyroglobulin.

### Treatment outcomes

**Figure 1:**
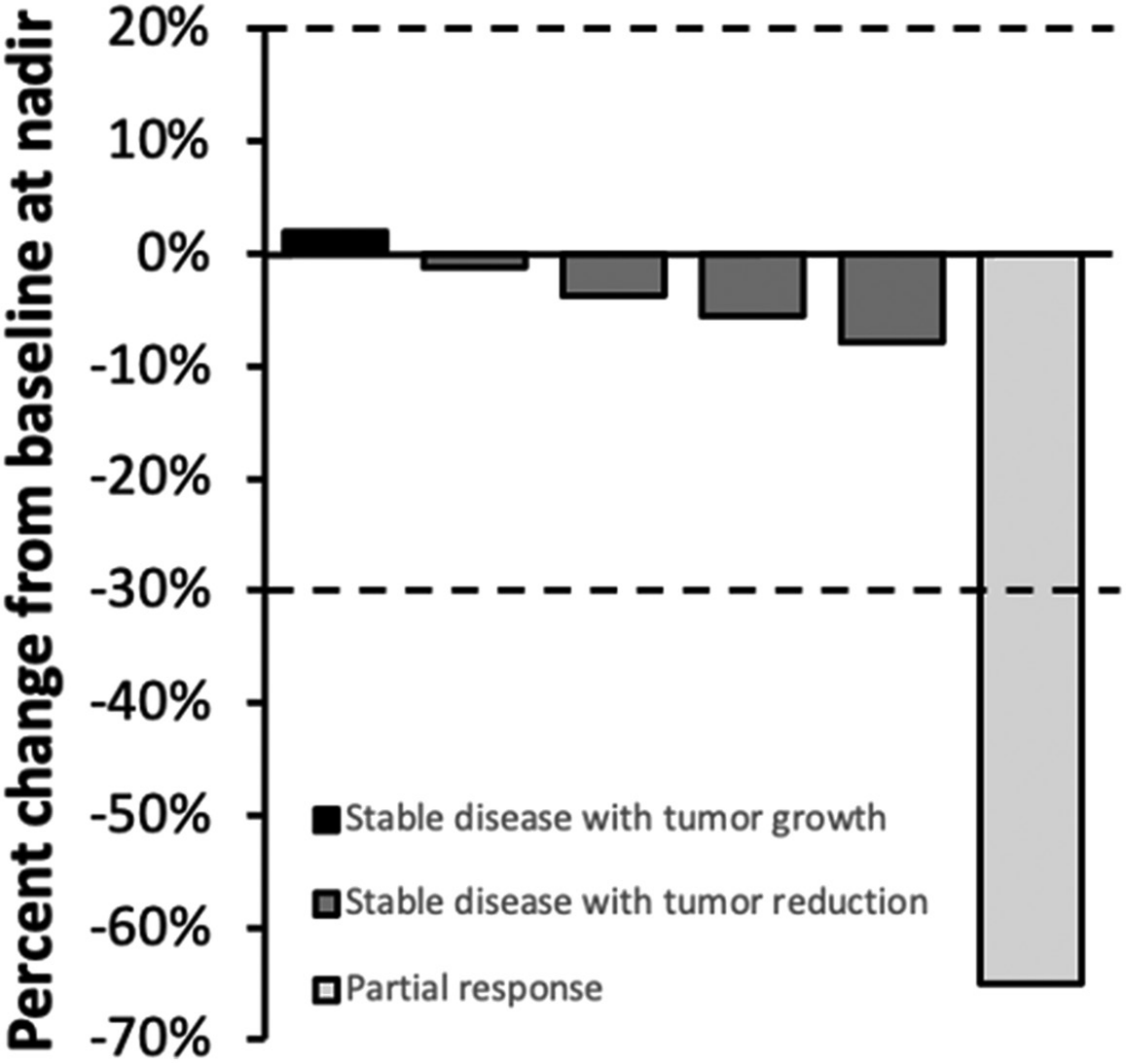
Waterfall chart of percentage change in the summed longest diameter of target lesions from baseline to nadir.

**Figure 2:**
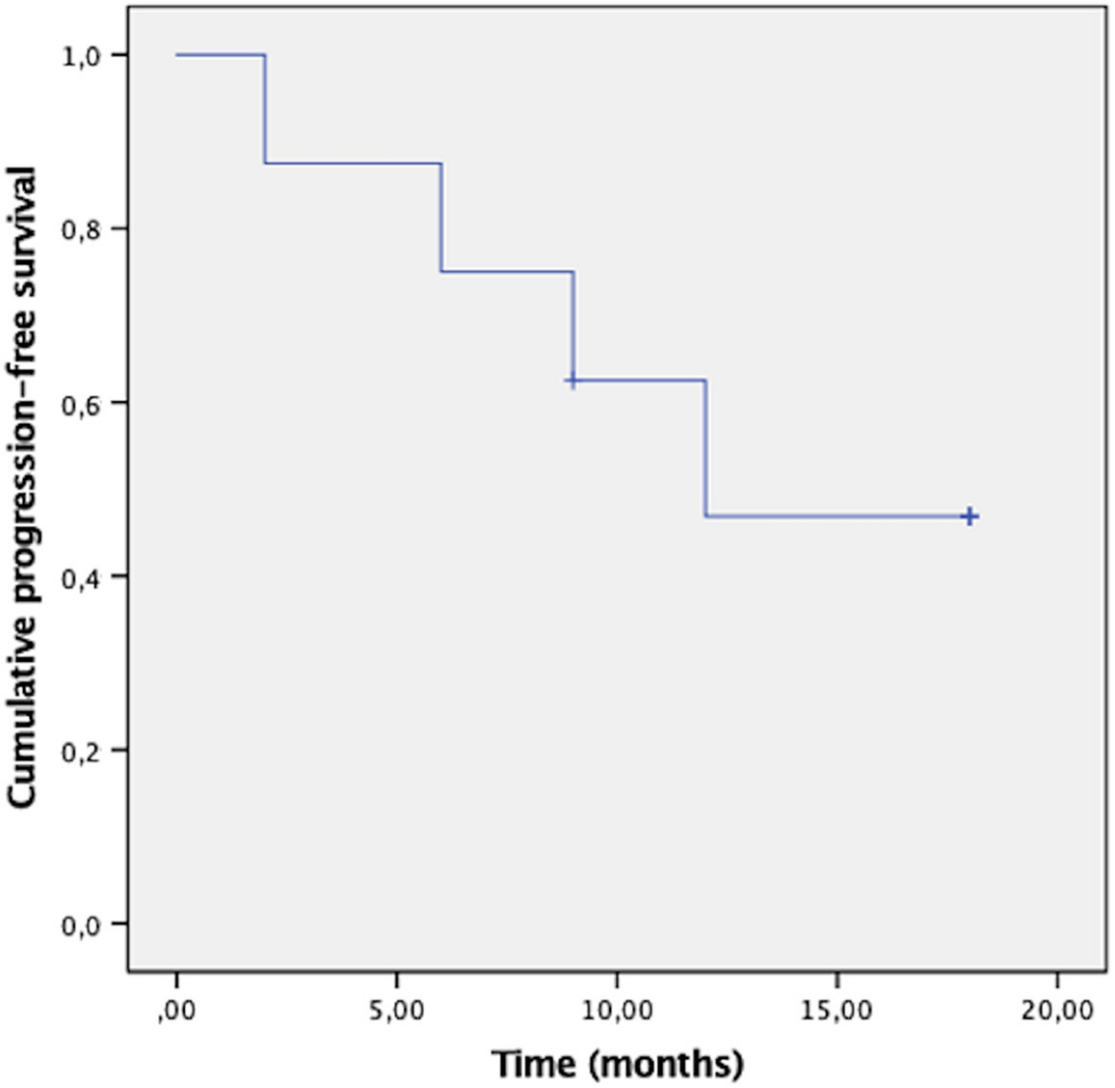
Kaplan–Meier chart for progression-free survival of the eight patients treated with lenvatinib for PDTC.

Details of lenvatinib treatment and effectiveness outcomes are listed in Table 3. The median duration of treatment was 10.3 months, and the median follow-up from lenvatinib start was 16.0 months. DCR was 100%, with a BOR of partial response in one patient (12.5%) and stable disease in seven patients (87.5%). As stable disease includes a wide range of tumor responses, Fig. 1 details BOR according to the variation of summed longest diameter of target lesions in each patient. Two patients have been monitored with PET-FDG, so they could not be included in this analysis. The median time to BOR was 3 months, and the median DoR was 7.5 months. The median PFS was 12.0 months (Fig. 2). The median OS was not reached since more than half of the patients were alive by the end of the study. OS at 3, 6, 12, and 18 months was 87.5, 87.5, 71.4, and 57.1%, respectively. Two patients continued lenvatinib beyond the evidence of tumor progression. However, subsequent evaluations would confirm further progression of the disease, and treatment was suspended 3 and 6 months later, respectively. The two patients with unresectable disease at lenvatinib start could not be submitted to surgery later, one due to rapid progression of the disease and the second due to unaltered dimensions of the primary tumor. By study end, three patients have died from disease-related causes. As such, in this study, DSS overlaps with OS. Five patients were still alive, one with partial response, three with stable disease, and one with disease progression.

**Table 3:** Details of lenvatinib treatment and effectiveness outcomes.

Outcome	Result
Median duration of treatment, months (range)	10.3 IQR 15.9 (1–19)
Median follow-up after treatment start, months (range)	16.0 IQR 11.8 (2–22)
Disease control rate, %	100
Best overall response, *n* (%)	
Partial response	1 (12.5)
Stable disease	7 (87.5)
Median time to best overall response, months (range)	3.0 IQR 0 (1–6)
Median duration of response, months (range)	7.5 IQR 12 (1–15)
Median progression-free survival, months	12.0
Median overall survival, months	Not reached
3 months, *n* (%)	7/8 (87.5)
6 months, *n* (%)	7/8 (87.5)
12 months, *n* (%)	5/7 (71.4)
18 months, *n* (%)	4/7 (57.1)
Median Tg in positive cases, at nadir, ng/mL	487.0 IQR 639.0 (36–907)
Median reduction of Tg in positive cases, at nadir, %	91 IQR 13 (81–97)
Median starting dose, mg (range)	17.0 IQR 13 (10–24)
24 mg, *n* (%)	3 (37.5)
20 mg, *n* (%)	1 (12.5)
14 mg, *n* (%)	2 (25)
10 mg, *n* (%)	2 (25)
Median average dose, mg	18.1 IQR 9.6 (13–24)

IQR, interquartile range; Tg, thyroglobulin.

### Lenvatinib dosage, adverse effects, and tolerability

The median initial dose was 17 mg/day and only three patients have started with 24 mg/day due to ECOG performance status, age, and comorbidities. The median average dose of lenvatinib was 18.1 mg/day, confirming the possibility to increment drug dose in some patients. Reduction of dose and temporary interruption of treatment were necessary in four (50%) and two (25%) patients, respectively, due to grade 1–2 AEs. Of those, two patients needed multiple reductions and temporary suspensions due to weight loss and hand–foot syndrome, respectively. The median time to first treatment reduction was 6.5 months. Lenvatinib-related AEs were experienced by 100% of patients, and a total of 11 different AEs were reported. The median time to the first AE was 37 days. The most frequent were fatigue (6 patients, 75.0%), weight loss (5 patients, 62.5%), anorexia (5 patients, 62.5%), hypertension (4 patients, 50.0%), and diarrhea (four patients, 50%). Fatigue was mainly managed with more time for rest and sleep, and avoidance of heavy physical activities. Adrenal insufficiency was investigated in selected cases and was not confirmed in any patient. Grade 3 AEs were reported in two patients: tracheoesophageal fistula and leg cellulitis. The former occurred in a 34-year-old woman who was first submitted to a first total thyroidectomy with lymphadenectomy in another hospital, and then submitted to new cervical surgery, 150 mCi RAI and neck EBRT at our institution 2 years later. Considering the rapid progression, a low dose of 14 mg/day was initiated. However, tracheoesophageal fistula would develop 2 months after lenvatinib start, leading to treatment suspension. A new progression of disease was evident shortly after and the patient eventually died 9 months after this event. The second patient was a 52-year-old man who was submitted to total thyroidectomy and lymphadenectomy and 150 mCi RAI 5 years before, has progressed over sorafenib, and showed stable disease for 18 months under lenvatinib. At this time, a diffuse itching cutaneous rash eventually emerged, evolving into right thigh cellulitis. Treatment with flucloxacillin was successful, but lenvatinib suspension was decided. He was under dexamethasone treatment for bone metastasis edema control, which may have facilitated the development of this complication. This patient was still alive at study-end, 9 months after this event. All reported AEs are detailed in Table 4.

**Table 4:** Adverse events during lenvatinib treatment.

Adverse events, *n* (%)	Total	Grade 1–2	Grade ≥ 3
Fatigue	6 (75.0)	6 (100)	0
Weight loss	5 (62.5)	5 (100)	0
Anorexia	5 (62.5)	5 (100)	0
Hypertension	4 (50.0)	4 (100)	0
Diarrhea	4 (50.0)	4 (100)	0
Oral mucositis	3 (37.5)	3 (100)	0
Proteinuria	2 (25.0)	2 (100)	0
Hand–foot syndrome	2 (25.0)	2 (100)	0
Cough	1 (12.5)	1 (100)	0
Tracheoesophageal fistula	1 (12.5)	0	1 (100)
Cellulitis	1 (12.5)	0	1 (100)

## Discussion

This study aimed to evaluate the real-world effectiveness and tolerability of lenvatinib in patients with PDTC. This represents a subgroup of RAI-refractory thyroid carcinomas with aggressive behavior and limited therapeutic options. As noted earlier, TKIs have been the most studied drugs in this setting, but specific results in PDTC patients are scarce. In the SELECT trial, lenvatinib elicited a PFS of 14.8 months (vs 2.1 with placebo) in PDTC patients (10). Since then, multiple real-world studies have also shown interesting results in RAI-refractory thyroid carcinoma patients, with PFS ranging from 10.0 to 35.3 months for both DTC and the minority of PDTC (11, 12, 13, 14, 15, 16, 17, 18). Our study has reached a PFS of 12 months, which is significant in a population with confirmed progressive disease and a worse expected prognosis compared to overall RAI-refractory thyroid carcinomas. However, comparisons of results between studies should be made carefully, due to different cohort characteristics. Our cohort had evidence of active disease progression, with a progression rate in the last 6 months of 100%. This is different from other real-world studies. Also, in the SELECT trial, patients were included if radiologic evidence of disease progression was evident within the last 13 months, eventually selecting a cohort with less aggressive disease (10). Concerning ECOG performance status, our population had considerably unfavorable ratios, with 50% with ECOG grade 2, compared to less than 5% in SELECT and 0–18% in other real-world studies (10, 11, 12, 13, 14, 15, 16). Compared to the mentioned studies, the median initial dose was lower, which could have had a negative impact on clinical results. This was decided mainly due to pre-treatment performance status and in an attempt to increase treatment tolerability. Noteworthy, the median average dose was slightly higher than the initial dose, which suggests good tolerability. Our study has also included a significant proportion of TKI pre-treated patients (37.5%), which suggests the presence of more advanced and drug-resistant diseases. All of our patients experienced at least one AEs, which is similar to previous reports. Similarly, fatigue, weight loss, anorexia, hypertension, and diarrhea were the most frequent AEs reported. The majority of this grade 1–2 AEs were managed without lenvatinib dose reductions or suspensions. One patient with significant weight loss had transient dose reductions and could then sustain stable weight under megestrol acetate. Also, two patients with very frequent diarrhea episodes were successfully managed with loperamide and intermittent reductions of lenvatinib dose. Finally, one patient with hand–foot syndrome had multiple dose reductions and interruptions every time symptoms recurred. Limitations to our study include the small cohort, which is in line with the rarity of the disease. The retrospective design could also impact our results. However, all patients were treated by the same multidisciplinary team and according to the same protocols, which limits variability. In conclusion, this study is the first to specifically confirm the real-world effectiveness of lenvatinib in PDTC patients. This applies to patients previously treated with conventional therapy and TKIs, and, also, to patients treated in the neoadjuvant setting.

## Declaration of interest

There is no conflict of interest that could be perceived as prejudicing the impartiality of the research reported.

## Funding

Ricardo Rodrigues was granted with a PhD scholarship by iNOVA4Health Research Unit (UIDP/04462/2020; UI/BD/154256/2022). The remaining authors did not receive any specific grant from funding agencies in the public, commercial, or non-for-profit sectors.

## Author contribution statement

All authors contributed to the study conception and design. All authors read and approved the final manuscript.
